# Pediatric defibrillation shocks alone do not cause heart damage in a porcine model

**DOI:** 10.1016/j.resplu.2022.100203

**Published:** 2022-02-01

**Authors:** Ben McCartney, Adam Harvey, Amy Kernaghan, Sara Morais, Olibhéar McAlister, Paul Crawford, Pardis Biglarbeigi, Raymond Bond, Dewar Finlay, David McEneaney

**Affiliations:** a Faculty of Computing, Engineering & Built Environment, Ulster University, Newtownabbey, United Kingdom^1^; b HeartSine Technologies Ltd., Stryker Belfast, Belfast, United Kingdom^2^; c Veterinary Anaesthesia Consultancy, Larne, Co. Antrim, United Kingdom^3^; d Department of Cardiology, Southern Health & Social Care Trust, Craigavon, United Kingdom^4^

**Keywords:** Paediatric, Defibrillation, AED, Waveforms, Resuscitation, Biomarkers

## Abstract

**Aim:**

Automated external defibrillators (AEDs) use various shock protocols with different characteristics when deployed in pediatric mode. The aim of this study is to assess and compare the safety and efficacy of different AED pediatric protocols using novel experimental approaches.

**Methods:**

Two defibrillation protocols (A and B) were assessed across two studies: Protocol A: escalating (50–75–90 J) defibrillation waveform with higher voltage, shorter duration and equal phase durations. Protocol B; non-escalating (50–50–50 J) defibrillation waveform with lower voltage, longer duration and unequal phase durations.

Experiment 1: Isolated shock damage was assessed following shocks to 12 anesthetized pigs. Animals were randomized into two groups, receiving three shocks from Protocol A (50–75–90 J) or B (50–50–50 J). Cardiac function, cardiac troponin I (cTnI), creatine phosphokinase (CPK) and histopathology were analyzed. Experiment 2: Defibrillation safety and efficacy were assessed through shock success, ROSC, ST-segment deviation and contractility following 16 randomized shocks from protocol A or B delivered to 10 anesthetized pigs in VF.

**Results:**

Experiment 1: No clinically meaningful difference in cTnI, CPK, ST-segment deviation, ejection fraction or histopathological damage was observed following defibrillation with either protocol. No difference was observed between protocols at any timepoint. Experiment 2: all defibrillation types demonstrated shock success and ROSC ≥ 97.5%. Post-ROSC contractility was similar between protocols.

**Conclusions:**

There is no evidence that administration of clinically relevant shock sequences, without experimental confounders, result in significant myocardial damage in this model of pediatric resuscitation. Typical variations in AED pediatric mode settings do not affect defibrillation safety and efficacy.

## Highlights

• AEDs utilize specific low energy pediatric modes to reduce myocardial damage.
• This study applied various shocks in sinus rhythm without cardiac instrumentation.
• Isolated clinically relevant shock sequences do not result in myocardial damage.
• Typical variations in pediatric shocks did not affect safety and efficacy.
• These results may inform future pediatric resuscitation guidelines.

## Graphical abstract

**Figure:**
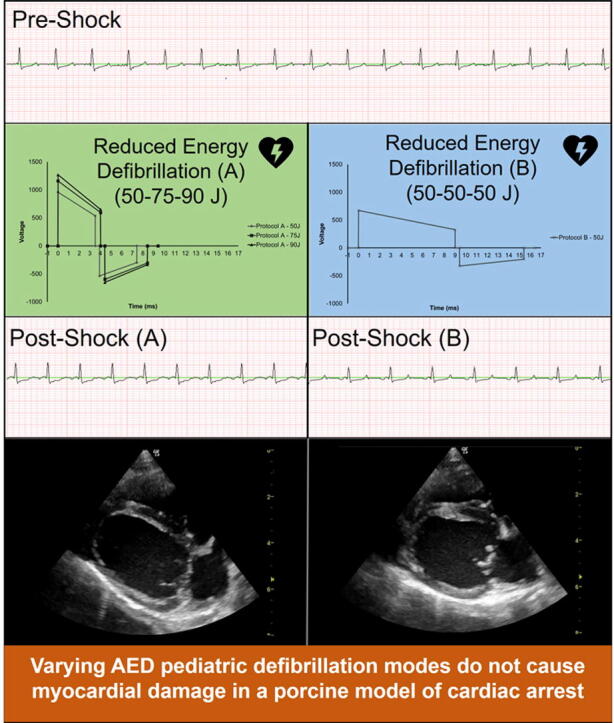

## Introduction

Pediatric patients account for approximately 2% of out-of-hospital cardiac arrest (OHCA).^1^, ^2^ Approximately 6.5–8% of pediatric OHCA patients present with shockable initial rhythms^1^, ^3^ significantly lower than that of adults (13.5%).^1^ Survival to hospital discharge from pediatric OHCA varies from approximately 2–6%.^4^, ^5^

The rarity of pediatric OHCA necessitates that pediatric defibrillation guidelines be extrapolated from adult arrest and animal studies.^6^ Recent publications highlight the lack of data on which to base pediatric energy dosage recommendations and the lowest effective dose, optimum dose and the upper limit for safe defibrillation are currently unknown.^7^ A real-world comparative waveform study is impractical due to the rarity of pediatric cardiac arrest. However, pediatric patients have been effectively defibrillated with a range of energies.^8^, ^9^ Pre-clinical studies demonstrate a wide safety margin for defibrillation.^10^

The European Resuscitation Council (ERC) guidelines recommend non-escalating doses of 4 J/kg while acknowledging doses < 9 J/kg have been used safely with negligible side effects.^11^ The American Heart Association (AHA) guidelines suggest energy doses of 2–4 J/kg.^12^ AHA and ERC align on classification of a pediatric patient as 1–8 years or 10–25 kg. Therefore, the recommended energy for a pediatric initial shock is 20–50 J, and 40–100 J if escalation occurs.

Public access automated external defibrillators (AEDs) are designed to be rapidly deployed by laypersons. To ensure uncomplicated use, weight-based dosing is replaced by pediatric specific modes. The configuration of AED pediatric modes varies with both escalating and non-escalating protocols and different waveforms. Removing the ability to tailor shock energy for the patient increases probability of delivering defibrillation dosages outside the recommended range. Despite the proliferation and increased knowledge of AEDs, reports of fear of use still persists.^13^

Biphasic defibrillation energy can be modified by altering current, voltage or phase duration (amount of time that current flows in a given direction) and many configurations exist at a given energy dose. As patient impedance increases, current will decrease, reducing energy delivered. Many AEDs compensate by increasing voltage and/or waveform duration.

There is no standard for biphasic waveforms and AEDs utilize various waveforms, featuring a range of currents, voltages and phase durations.^14^ Biphasic defibrillation, which features reduced current and voltage compared to monophasic defibrillation, results in better post-resuscitation cardiac function.^15^ It is unknown if differences in current, voltage and duration between biphasic waveforms affect safety or efficacy.^16^, ^17^

Defibrillation protocols in modern AEDs utilize various energies and waveform characteristics. Despite the range of biphasic waveforms available, it has been suggested that the best AED is the first available,^18^ even without a pediatric mode.^19^ The safety and efficacy of distinct waveforms, with contrasting configurations, that appear suitable for pediatric patients have never been directly compared.

Previous research typically applied shocks after cardiac instrumentation and/or ventricular fibrillation (VF) induction, confounding interpretation of resulting myocardial injury.^20^, ^21^ This study applied shocks in sinus rhythm without cardiac instrumentation, isolating the effect on myocardium of shocks (experiment 1), significantly progressing understanding of pediatric defibrillation safety.

The objective of this study was to compare the safety and efficacy of two typical but notably different pediatric defibrillation protocols. These are representative of current variation in pediatric defibrillation waveforms and their deployment i.e. escalating and non-escalating.

## Methods

**Fig. 1:**
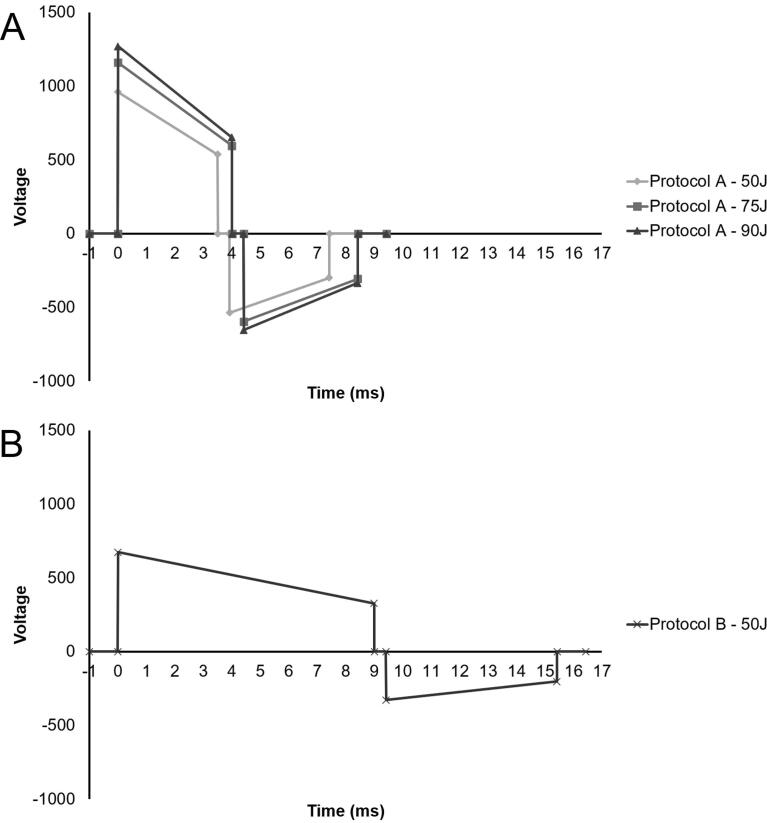
Biphasic defibrillation waveforms. A) Protocol A (50 J), Protocol A (75 J), Protocol A (90 J). B) Protocol B (50 J).

The two defibrillation protocols are described below (Fig. 1A-B):

*Protocol A*: an escalating protocol of 50–75–90 J. To deliver 50 J at 50 Ohms patient impedance, this waveform features a peak voltage of 960 volts and a duration of 7.4 ms. The duration of phase 1 and 2 are equal.

*Protocol B*: a non-escalating protocol of 50–50–50 J. To deliver 50 J at 50 Ohms patient impedance, this waveform features a peak voltage of 673 volts and a duration of 15.4 ms. The duration of phase 1 is greater than phase 2.

**Fig. 2:**
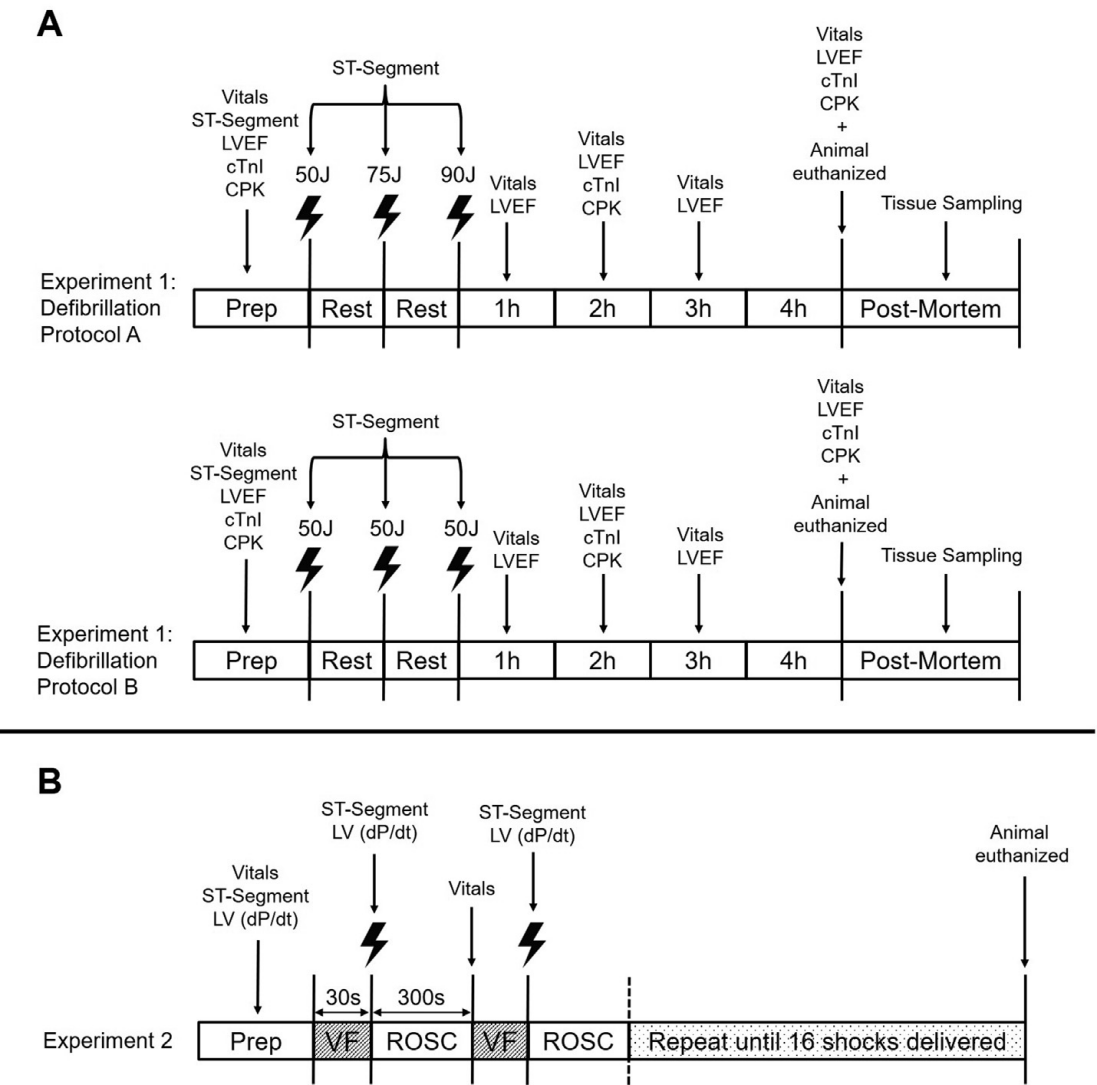
Timelines for A) Experiment 1 and B) Experiment 2. VF- ventricular fibrillation, ROSC- return of spontaneous circulation. Vitals- Vital signs (blood pressure, end-tidal carbon dioxide, peripheral saturation of oxygen), ST-Segment- ST-segment displacement from baseline, LV dP/dt- change in rate of change of LV pressure from baseline, LVEF- left ventricular ejection fraction, cTnI- cardiac troponin I, CPK- creatine phosphokinase, 1–4 h-1–4-hours post-shock.

Due to difficultly measuring both safety and efficacy in a single study, a two-experiment design was utilized (Fig. 2A-B).

## Subjects

Studies were carried out according to Animals (Scientific Procedures) Act 1986 and approved by University of Edinburgh Animal Welfare and Ethical Review Body. Experiments were performed on commercial female Large White Landrace cross or Large White Landrace Duroc cross swine (7–9 weeks, 10–23 kg) deemed to be in good health by qualified personnel. Animals were sedated and anesthetized. A surgical approach was made to blood vessels requiring cannulation. Further details are found in Supplementary Material: Supplemental methods. 3-lead ECG, arterial blood pressure, end-tidal carbon dioxide (EtCO_2_), were measured using an S/5 monitor (Datex Ohmeda, Madison, USA), regional oximetry (rSO_2_) was recorded using an INVOS 5100C (Medtronic, Minneapolis, USA) (Experiment 1 only). A samaritan PAD 350P AED (HeartSine, Belfast, UK), programmed with either Protocol A or B was attached using electrodes placed in the anterior-posterior position, delivered shocks. ST-segment deviation was calculated from lead 2 of the 3-lead ECG using the ECG Analysis Module in LabChart Pro version 8 (AD Instruments, Oxford, UK) pre-shock, and at 10- and 60-seconds post-shock. Data is reported in accordance with ARRIVE 2.0 guidelines.

## Experiment 1 methods

Twelve swine (10–13 kg) were studied to directly assess shock-induced myocardial damage at energy doses of 3.8–9.6 J/kg. See Fig. 2A for timeline. Animals were randomly assigned a defibrillation protocol, six per group. A cannula was inserted into the saphenous artery and connected to a pressure transducer with a fluid-filled line.

## Shock delivery

Protocol A delivered a cumulative energy of 215 J (50 + 75 + 90 J) and Protocol B delivered a cumulative energy of 150 J (50 + 50 + 50 J). Animals then entered a 4-hour rest period.

## Measurements

Blood gas, electrolytes, and cardiac troponin I (cTnI) were assessed in heparinized whole blood (i-STAT 1 300, Abaxis, Union City, USA) pre-intervention, and 2-hours and 4-hours post-shocks. Complete blood count and serum creatine phosphokinase (CPK) were assessed using an Advia 2120 (Siemens Healthineers, Erlangen, Germany) and an AU480 biochemistry analyzer (Beckman Coulter, Brea, USA) pre-intervention, and 2-hours and 4-hours post-shockss.

B-mode echocardiographic images were acquired using a Logiq R7 (General Electric, Boston, USA) with 6S-RS probe (General Electric, Boston, USA) pre-intervention, and every hour post-shocks.

Tissue sections were dissected from the right ventricle (RV), left ventricle (LV), right atria (RA), left atria (LA), and lungs after the animal was euthanized post-rest period. Severity of tissue damage was evaluated for hemorrhage, inflammation, thrombi, and necrosis according to a standard scoring system; none (0), mild (1), moderate (2) and severe (3).^22^

## Experiment 2 methods

The safety and efficacy of individual shocks within each protocol was studied in 10 swine (12–23 kg). See Fig. 2B for timeline. Mikro-Tip catheters (Millar, Houston, USA) were delivered via carotid arteries to measure LV and aortic pressures. VF was electrically induced as previously described^23^ and left untreated for approximately 30-seconds before defibrillation.

## Shock delivery

Each animal received 16 single shocks randomly selected from the 4 different shock types (Protocol at 50, 75 or 90 J or Protocol B at 50 J). This totalled 160 shocks across the study, with 40 observation for each of the 4 shock type groups. Following ROSC, animals entered an approximately 5-minute rest period, after which VF was re-induced and the next shock delivered. If ROSC was not achieved 30-seconds post-shock, CPR and 150 J shocks were delivered until ROSC or until the third ECG analysis. If ROSC was not achieved the animal was euthanized. If ROSC was achieved, the protocol resumed.

## Measurements

The difference in rate of change of left ventricular pressure (LV dP/dt) was calculated from the LV pressure for 180 seconds post-shock using LabChart Pro 8 (AD Instruments, Oxford, UK).

### Data analysis

Pre-intervention values were summarized with medians and interquartile ranges. Boxplots were used to present cTnI, CPK, LVEF, ST-Segment, time to first perfusing beat and time to sinus rhythm data. Bar charts were used to present shock success and ROSC data. A line chart was used to present LV dP/dt data.

The following statistical methods were used for each analysis. Experiment 1, all analyses: Mann-Whitney Test. Experiment 2, ST-segment deviation: LV dP/dt: one-way ANOVA with Tukey comparison, shock success, ROSC: Fishers Exact Test, Time to first perfusing beat, time to sinus rhythm: Kaplan-Meier survival analysis. The methods were deemed appropriate by use of normality tests. Statistical significance was denoted in figures and tables if observed. Analyses were conducted by staff blinded to group allocation as appropriate using Minitab 19, R version 3.6.1 or Microsoft Excel 2008. p ≤ 0.05 was considered statistically significant.

## Results

### Experiment 1 results

An equipment failure in experiment 1 led to one animal from the Protocol B group being excluded from the analysis after pre-intervention data was collected. Three-lead ECG data was unavailable for 1 animal from each group due to recording failure.

Pre-intervention parameters are summarized in Table 1, animals assigned to both groups displayed normal physiology, including blood pressure and temperature, and no significant differences were observed.

**Table 1:** Experiment 1 pre-intervention parameters.

**Parameter**	**Defibrillation protocol**	**Median**	**IQR**	**P value**
Weight (kg)	Protocol A	12.00	1.25	1.000
	Protocol B	12.00	1.13	
Heart Rate (BPM)	Protocol A	107.50	34.00	0.810
	Protocol B	105.50	40.00	
Ventilation Rate (VPM)	Protocol A	28.00	4.75	0.298
	Protocol B	31.00	9.00	
Systolic Blood Pressure (mmHg)	Protocol A	93.50	20.25	0.378
	Protocol B	101.00	15.75	
Diastolic Blood Pressure (mmHg)	Protocol A	57.00	18.75	0.749
	Protocol B	57.50	11.00	
Temperature (°C)	Protocol A	36.85	0.70	0.173
	Protocol B	37.30	0.80	
Regional saturation of oxygen (%)	Protocol A	49.00	15.25	0.173
	Protocol B	46.50	7.00	
EtCO_2_ (mmHg)	Protocol A	44.50	11.75	1.000
	Protocol B	45.00	8.50	
cTnI (ng/mL)	Protocol A	0.02	0.03	1.000
	Protocol B	0.02	0.04	
CPK (U/L)	Protocol A	279.00	241.30	1.000
	Protocol B	285.50	136.30	
LVEF (%)	Protocol A	60.06	9.50	0.936
	Protocol B	58.68	6.78	

N = 6 animals. EtCO_2_-End-Tidal carbon dioxide, cTnI -Cardiac troponin I, CPK- Creatinine phosphokinase, LVEF -Left ventricular ejection fraction. IQR- Interquartile range.

**Fig. 3:**
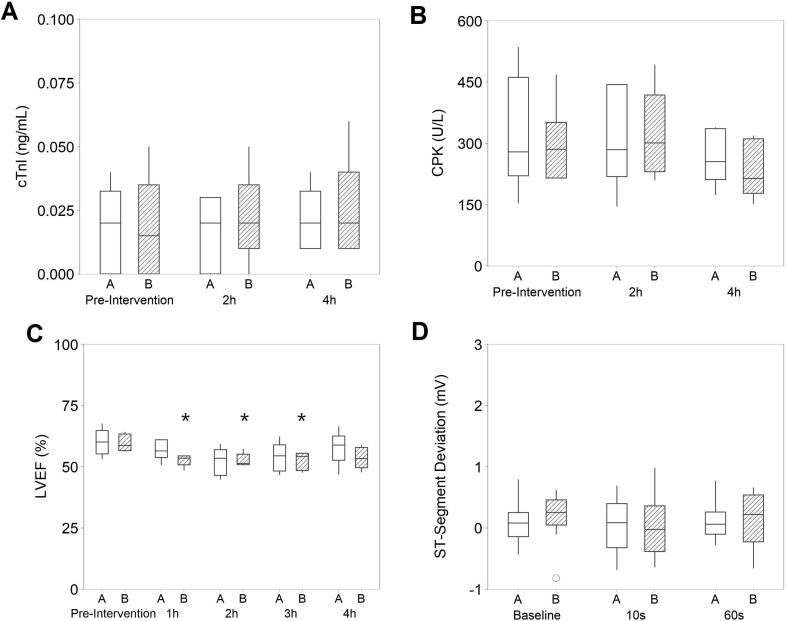
Cardiac damage assessment following shocks from protocols A and B. A) cTnI (ng/mL) at baseline, 2-hours and 4-hours post-shock. B) CPK (U/L) at baseline, 2-hours and 4-hours post-shock. C) LVEF (%) at baseline, 2-hours and 4-hours post-shock. D) ST-segment deviation (mV) at baseline, 10-seconds post-shock and 60-seconds post-shock. A-C: N = 5–6 animals. D: N = 4–6 animals. cTnI -Cardiac troponin I, CPK- Creatinine phosphokinase, LVEF -Left ventricular ejection fraction. * p < 0.05 vs pre-intervention.

Biomarkers for cardiac injury, cTnI and CPK, were assessed at 2- and 4-hours post-delivery of the final shock. No difference was observed for either biomarker at any timepoint, compared to baseline, and there were no significant differences between protocols (Fig. 3A-B). LVEF did not differ from baseline for protocol A at any timepoint. Conversely 1, 2 and 3-hour LVEF results were significantly reduced from baseline for protocol B, while 4-hour values were not significantly different to baseline values (Fig. 3C). ST-segment deviation at 10- and 60-seconds post-shock was not statistically different baseline and there were no significant differences between protocols, (Fig. 3D).

Additional blood parameters were measured at pre-intervention and 2-hours and 4-hours post-defibrillation. Supplemental Table 1 summarizes additional blood parameters. Histological analysis was conducted on heart and lungs tissue samples. Supplemental Table 2 summarizes histological analysis. No significant differences between groups were observed for any tissue sample in hemorrhage, inflammation, thrombosis or necrosis. Median values for all parameters were below moderate levels.

### Experiment 2 results

Experiment 1 demonstrated that AED shocks alone did not produce myocardial damage in the pediatric model. Furthermore, there were no significant differences between protocols. Experiment 2 was designed to further compare protocols in terms of efficacy, performance and acute safety.

Due to recording failure in experiment 2, ECG data was unavailable for 1 animal, whilst 2 animals had partial recordings. This resulted in 33, 32, 30 and 33 ST-segment deviation observations following successful defibrillation for Protocol A: 50, 75 and 90 J and Protocol B 50 J respectively. Additionally, LV pressure data for 2 shocks was not available. This resulted in 39, 39, 40, 40 LV pressure observations for Protocol A: 50, 75 and 90 J and Protocol B 50 J respectively.

Each animal received 16 randomized shocks from both defibrillation protocols (Protocol A: 50, 75 or 90 J, Protocol B: 50 J) following VF-induction.

Pre-intervention parameters are listed in Table 2.

**Table 2:** Experiment 2 pre-intervention parameters.

**Parameter**	**Median**	**IQR**
Weight (kg)	16.50	6.00
Heart Rate (BPM)	102.00	16.00
Ventilation Rate (VPM)	27.00	2.00
Systolic Blood Pressure (mmHg)	93.50	11.00
Diastolic Blood Pressure (mmHg)	48.50	8.00
Temperature (°C)	37.65	0.40
EtCO_2_ (mmHg)	43.50	2.00
ST-Segment Deviation (mV)	0.65	0.24
LV dP/dt (maximum) (mmHg/s)	1090.53	322.23

N = 9–10 animals. EtCO_2_-End-Tidal carbon dioxide), LV dP/dt- rate of change of left ventricular pressure. IQR- Interquartile range.

**Fig. 4:**
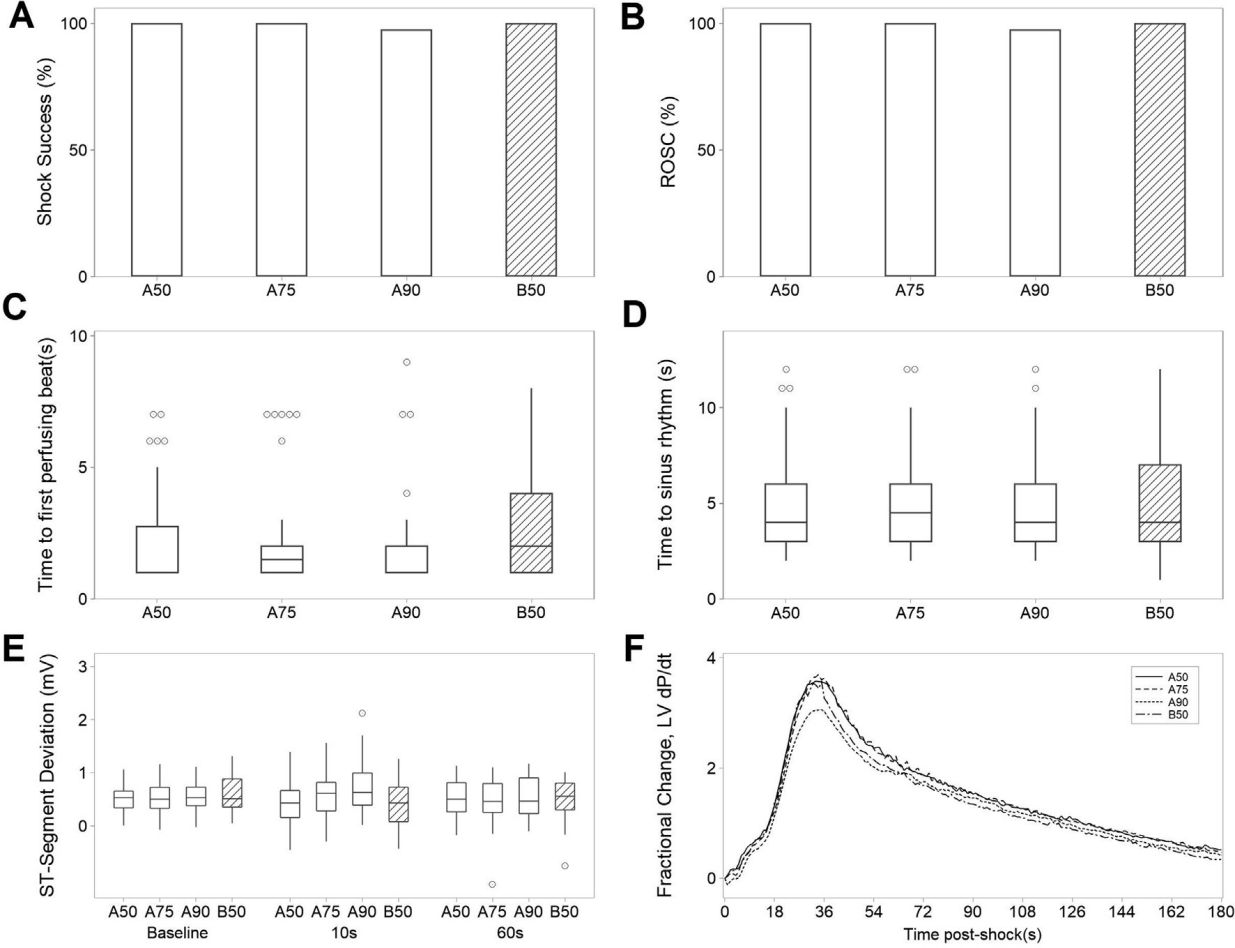
Assessment of the efficacy and safety of defibrillation types. A) Proportion of shock success (%) for each type B) Proportion of shocks with ROSC (%) C) time to first perfusing beat (seconds) post-defibrillation D) time to sinus rhythm (seconds) post-defibrillation. E) ST-segment deviation (mV) at baseline, 10- and 60-seconds post-successful defibrillation. F) Fractional change, LV dP/dt from baseline for 180-seconds post-defibrillation A-D: N = 10 animals, n = 40 shocks. E: N = 9 animals, n = 30–33 shocks. F: N = 10 animals, n = 39–40 shocks. A50/A75/A90- defibrillation Protocol A at 50/75/90 J, B50- defibrillation Protocol B at 50 J.

All defibrillation types demonstrated high levels of shock success and ROSC (Fig. 4A-B). All individual shocks but one (Protocol A-90 J) were successful and resulted in ROSC. No difference was observed in time until first perfusing beat and time until sinus rhythm between defibrillation types (Fig. 4C-D).

Safety was assessed through ST-segment deviation at 10- and 60-seconds post-shock and LV dP/dt at 1-, 10- and 60-seconds post-shock. There were no significant differences between defibrillation types in ST-segment deviation at any timepoint (Fig. 4E). Peak median LV dP/dt occurred at 35-seconds post-defibrillation. No significant differences were observed at this timepoint (Fig. 4F). Area under the curve was calculated for LV dP/dt for 180-seconds post-shock; no significant differences were found (Fig. 4F).

## Discussion

This study demonstrates that isolated shocks from two pediatric biphasic defibrillation protocols do not cause myocardial damage and both protocols were highly efficacious.

In Experiment 1 shocks were delivered in sinus rhythm, removing potential for damage from VF-induction or cardiac instrumentation^20^, ^21^ and thus directly assesses potential for myocardial damage caused by shocks. Cardiac damage biomarkers, LVEF and ST-segment deviation were compared with baseline. Cardiac biomarkers and ST-segment deviation were not significantly different to baseline at any timepoint. LVEF results did not differ from baseline for protocol A. Conversely 1–3-hour LVEF results were significantly different from baseline for protocol B. Values were however within 8% of baseline at every timepoint. Additionally, all median values were comparable to normal porcine LVEF values for anesthetized pigs,^24^ indicating a lack of clinical significance.

Comparisons were also made between groups. There were no significant differences in cardiac damage biomarkers, LVEF or ST-segment deviation between defibrillation protocols, indicating waveform variations did not affect safety. Previously, shocks after VF-induction have been shown to cause cTnI elevation.^22^ ST-segment deviation occurs when myocardial damage or ischemia is present^25^ and may occur as early as 1–3 seconds post-defibrillation.^25^, ^26^ Here, ST-segment deviation assessed at 10 and 60-seconds post-sinus rhythm shocks were similar in both groups.

Histopathological damage has been reported following defibrillation.^22^ Samples revealed no significant damage or differences between defibrillation protocols for hemorrhage, inflammation, thrombosis and necrosis. Surprisingly, low median levels of necrosis were observed in both groups in the left ventricle only. This finding is difficult to comprehend, given the absence of cardiac damage biomarker elevation, inflammation, thrombosis and hemorrhage. Experiment 2 focused on acute safety and efficacy of individual shocks, comparing rates of shock success and ROSC. It has been reported that pediatric defibrillation doses of 2 J/kg are inadequate for children closer to 25 kg.^27^ In this study, all defibrillation types (2.2–7.5 J/kg) demonstrated high levels of shock success and ROSC ≥ 97.5%. No difference in time until first perfusing beat and time until sinus rhythm was observed between groups.

This study reports higher defibrillation efficacy than similar studies^28^, ^29^ possibly due to short VF duration. High levels (90–100%) of shock success have been reported after 15-seconds^30^ and 30-seconds^31^ post-VF-induction. At longer durations (2–7 minutes), shock success reduces despite a high instance of shockable rhythms.^28^, ^29^, ^32^ This may be a consequence of cumulative cardiac damage ensuing from ischemia, cardiac instrumentation and CPR.

Safety was assessed through analysis ST-segment deviation and LV contractility.^33^ No significant differences in ST-segment deviation were observed between protocols at any timepoint. Differences in ST-segment deviation at 10-seconds post-shock when comparing monophasic and biphasic waveforms have been reported.^34^ Prolonged or persistent ST-segment deviation is a key indicator of cardiac ischemia.^35^

Impaired contractility following resuscitation from SCA has been reported.^36^ Contractile and hemodynamic changes following defibrillation are often transient with increased damage being associated with prolonged contractile dysfunction.^37^ In this study there was no difference in myocardial contractility between defibrillation types, indicating comparable safety and typically returned to baseline levels within 5-minutes post-shock.

Significantly reduced cardiac function in pediatric porcine models following attenuated adult biphasic, monophasic weight-based shocks^28^ and adult shocks^29^ has been reported. This was not observed in the present study, most likely a result of study design variation. We delivered shocks to animals in sinus rhythm (Experiment 1). The reduction in LVEF reported elsewhere is thus likely a result of cardiac instrumentation, VF-induction, ischemia, CPR where administered, or a combination. The previous study demonstrated differences in cardiac function between two pediatric defibrillation types in larger animals,^28^ indicating a potential interaction between ischemic downtime and defibrillation energy. The influence of defibrillation type on safety may be more pronounced after prolonged arrest.

This study has the following limitations. The extent of the transability of this pre-clinical research to humas sudden cardiac arrest is yet to be fully determined. Delivery of shocks in sinus rhythm removed confounding effects of instrumentation, VF-induction and ischemia and allowed for focused analysis of defibrillation-related myocardial damage. However, clinical translation will be difficult where extent of prior heart disease and resuscitation efforts will have effect. Defibrillation doses aligned with typical public access AED child mode settings, applicability to where weight-based defibrillation energies are utilized is limited. Clinically, diagnosing ischemia requires ST-segment deviation on 2 contiguous precordial leads or at least 2 adjacent limb leads of the 12-lead ECG.^38^ The single lead ST-segment recordings used here and elsewhere^25^ has reduced sensitivity and specificity for ischemia compared with 12-lead ECG recording.

## Conclusions

Administration of clinically relevant shock sequences isolated from the confounding influence of intracardiac instrumentation, VF induction, ischemia and CPR does not result in myocardial damage in this porcine model of pediatric resuscitation. Typical variations in biphasic waveforms in AEDs do not affect safety and efficacy in this model of pediatric cardiac arrest.

### CRediT authorship contribution statement

**Ben McCartney:** Conceptualization, Data curation, Formal analysis, Investigation, Methodology, Visualization, Writing – original draft, Writing – review & editing. **Adam Harvey:** Conceptualization, Investigation, Methodology, Supervision, Writing – review & editing. **Amy Kernaghan:** Conceptualization, Data curation, Formal analysis, Investigation, Methodology, Writing – review & editing. **Sara Morais:** Conceptualization, Data curation, Formal analysis, Investigation, Methodology, Writing – review & editing. **Olibhéar McAlister:** Conceptualization, Methodology, Writing – review & editing. **Paul Crawford:** Investigation, Methodology, Writing – review & editing. **Pardis Biglarbeigi:** Methodology, Supervision, Writing – review & editing. **Raymond Bond:** Methodology, Supervision, Writing – review & editing. **Dewar Finlay:** Methodology, Supervision, Writing – review & editing. **David McEneaney:** Conceptualization, Methodology, Supervision, Writing – review & editing.

## Declaration of Competing Interest

The authors declare the following financial interests/personal relationships which may be considered as potential competing interests: BM, AH, AK, SM and OM are employed by HeartSine Technologies Ltd., Stryker Belfast. PC is a consultant paid by HeartSine Technologies Ltd., Stryker Belfast. DM sits on the HeartSine Technologies Ltd., Stryker Belfast Clinical Advisory Board and is provided remuneration (modest).

## References

[1] Benjamin E.J., Muntner P., Alonso A. (2019). Heart Disease and Stroke Statistics—2019 Update: A Report From the American Heart Association. Circulation.

[2] Engdahl J., Axelsson Å., Bång A., Karlson B.W., Herlitz J. (2003). The epidemiology of cardiac arrest in children and young adults. Resuscitation.

[3] Matsui S., Kitamura T., Sado J. (2019). Location of arrest and survival from out-of-hospital cardiac arrest among children in the public-access defibrillation era in Japan. Resuscitation.

[4] Fink E.L., Prince D.K., Kaltman J.R. (2016). Unchanged pediatric out-of-hospital cardiac arrest incidence and survival rates with regional variation in North America. Resuscitation.

[5] Atkins D.L., Everson-Stewart S., Sears G.K. (2009). Epidemiology and Outcomes From Out-of-Hospital Cardiac Arrest in Children. Circulation.

[6] Haskell S.E., Atkins D.L. (2010). Defibrillation in children. J Emerg Trauma Shock.

[7] Mercier E., Laroche E., Beck B. (2019). Defibrillation energy dose during pediatric cardiac arrest: Systematic review of human and animal model studies. Resuscitation.

[8] Gurnett C.A., Atkins D.L. (2000). Successful use of a biphasic waveform automated external defibrillator in a high-risk child. Am J Cardiol.

[9] Rossano J.W., Quan L., Kenney M.A., Rea T.D., Atkins D.L. (2006). Energy doses for treatment of out-of-hospital pediatric ventricular fibrillation. Resuscitation.

[10] Babbs C.F., Tacker W.A., VanVleet J.F., Bourland J.D., Geddes L.A. (1980). Therapeutic indices for transchest defibrillator shocks: Effective, damaging, and lethal electrical doses. Am Heart J.

[11] Maconochie I.K., Bingham R., Eich C. (2015). European Resuscitation Council Guidelines for Resuscitation 2015: Section 6. Paediatric life support. Resuscitation.

[12] Topjian A.A., Raymond T.T., Atkins D. (2020). Part 4: Pediatric Basic and Advanced Life Support: 2020 American Heart Association Guidelines for Cardiopulmonary Resuscitation and Emergency Cardiovascular Care. Circulation.

[13] Bogle B., Mehrotra S., Chiampas G., Aldeen A.Z. (2013). Assessment of knowledge and attitudes regarding automated external defibrillators and cardiopulmonary resuscitation among American University students. Emerg Med J.

[14] Nichol G., Sayre M.R., Guerra F., Poole J. (2017). Defibrillation for Ventricular Fibrillation. J Am Coll Cardiol.

[15] Tang W., Weil M.H., Sun S. (2001). A Comparison of Biphasic and Monophasic Waveform Defibrillation After Prolonged Ventricular Fibrillation. Chest.

[16] Shan Y., Ristagno G., Fuller M. (2010). The effects of phase duration on defibrillation success of dual time constant biphasic waveforms. Resuscitation.

[17] Nanthakumar K., Newman D., Paquette M., Dorian P. (2005). Systematic evaluation of the determinants of defibrillation efficacy. Hear Rhythm.

[18] Niemann J.T. (2001). Defibrillation waveforms. Ann Emerg Med.

[19] Van de Voorde P., Turner N.M., Djakow J. (2021). European Resuscitation Council Guidelines 2021: Paediatric Life Support. Resuscitation.

[20] Alehan D., Ayabakan C., Çeliker A. (2003). Cardiac troponin T and myocardial injury during routine cardiac catheterisation in children. Int J Cardiol.

[21] Furniss G., Shi B., Jimenez A., Harding S.A., Larsen P.D. (2015). Cardiac troponin levels following implantable cardioverter defibrillation implantation and testing. Europace.

[22] Huang J., Ruse R.B., Walcott G.P. (2019). Ascending Defibrillation Waveform Significantly Reduces Myocardial Morphological Damage and Injury Current. JACC Clin Electrophysiol.

[23] Howe A., O’Hare P., Crawford P. (2015). An investigation of thrust, depth and the impedance cardiogram as measures of cardiopulmonary resuscitation efficacy in a porcine model of cardiac arrest. Resuscitation.

[24] Paslawska U., Noszczyk-Nowak A., Paslawski R. (2014). Normal electrocardiographic and echocardiographic (M-mode and two-dimensional) values in Polish Landrace pigs. Acta Vet Scand.

[25] Killingsworth C.R., Walcott G.P., Melnick S.B. (2002). Defibrillation threshold and cardiac function using an external biphasic defibrillator in pediatric-sized pigs. J Am Coll Cardiol.

[26] Gurevitz O., Lipchenca I., Yaacoby E. (2002). ST-Segment Deviation Following Implantable Cardioverter Defibrillator Shocks: Incidence, Timing, and Clinical Significance. Pacing Clin Electrophysiol.

[27] Tibballs J., Carter B., Kiraly N.J., Ragg P., Clifford M. (2011). External and internal biphasic direct current shock doses for pediatric ventricular fibrillation and pulseless ventricular tachycardia*. Pediatr Crit Care Med.

[28] Berg R.A., Chapman F.W., Berg M.D. (2004). Attenuated adult biphasic shocks compared with weight-based monophasic shocks in a swine model of prolonged pediatric ventricular fibrillation. Resuscitation.

[29] Berg R.A., Samson R.A., Berg M.D. (2005). Better outcome after pediatric defibrillation dosage than adult dosage in a swine model of pediatric ventricular fibrillation. J Am Coll Cardiol.

[30] Sullivan J.L., Melnick S.B., Chapman F.W., Walcott G.P. (2007). Porcine defibrillation thresholds with chopped biphasic truncated exponential waveforms. Resuscitation.

[31] Ristagno G., Yu T., Quan W., Freeman G., Li Y. (2012). Comparison of defibrillation efficacy between two pads placements in a pediatric porcine model of cardiac arrest. Resuscitation.

[32] Zhou Z., Wang Y., Zhou H. (2010). Defibrillation and resuscitation in a piglet model of pediatric ventricular fibrillation following AHA 2005 guidelines. Indian J Pediatr.

[33] Killingsworth C.R., Melnick S.B., Chapman F.W. (2002). Defibrillation threshold and cardiac responses using an external biphasic defibrillator with pediatric and adult adhesive patches in pediatric-sized piglets. Resuscitation.

[34] Reddy R.K., Gleva M.J., Gliner B.E. (1997). Biphasic Transthoracic Defibrillation Causes Fewer ECG ST-Segment Changes After Shock. Ann Emerg Med.

[35] Inohara T., Alfadhel M., Starovoytov A., John M.G., Saw J. (2020). Differences in revascularization strategy and outcomes in ST-elevation and non-ST-elevation myocardial infarction with spontaneous coronary artery dissection. Eur Heart J.

[36] Müllner M., Domanovits H., Sterz F. (1998). Measurement of myocardial contractility following successful resuscitation: quantitated left ventricular systolic function utilising non-invasive wall stress analysis. Resuscitation.

[37] Walcott G.P., Melnick S.B., Killingsworth C.R., Ideker R.E. (2010). Comparison of Low-Energy Versus High-Energy Biphasic Defibrillation Shocks Following Prolonged Ventricular Fibrillation. Prehospital Emerg Care.

[38] Antman E.M., Anbe D.T., Armstrong P.W. (2004). ACC/AHA guidelines for the management of patients with ST-elevation myocardial infarction - Executive summary: A report of the American College of Cardiology/American Heart Association Task Force on Practice Guidelines. Circulation.

