# Supplementary material for: Pediatric defibrillation shocks alone do not cause heart damage in a porcine model
Source: Resusc Plus. 2022 Feb 1;9:100203. doi: 10.1016/j.resplu.2022.100203 (PMC8816722; doi:10.1016/j.resplu.2022.100203)
Supplement: Supplementary Data 1 [file mmc1.docx]

**Supplementary Material**

**Supplemental Methods**

A porcine model was chosen as it produces a good replication of the human cardiovascular system. Animals were housed in pens with straw bedding in groups of at least two animals. Animals had access to food and water prior to commencement of the study. Animals which were determined to be in poor health, or outside of the pre-defined weight range were excluded. Inclusion and exclusion criteria were set before the study was conducted. All animals were under veterinary supervision from study commencement, including the time when the animals are under anaesthesia. Following sedation, animals were moved to a preparation room where they were intubated, after which they were moved to a surgery where the experiment was performed. No animals exhibited evidence of disease, ill health, distress or discomfort. Core body temperature was maintained between 36-39°C

Echocardiographic images were made on a right parasternal long axis four-chamber view and were analysed using the modified Simpsons method of disks. Left ventricular ejection fraction (LVEF) values were averaged from 3 separate LVEF calculations made at each timepoint in a blinded manner.

After completion of each experiment, the animal was then euthanized by a Schedule 1 Method, overdose of anaesthesia. No animals were excluded from the study. An experimental unit was defined as one animal. Control or untreated groups were not utilized in this study as the scope was to compare safety and efficacy of two distinct protocols. Randomisation schedules were generated using Microsoft Excel. To further minimize potential confounding factors, the animal test system was characterized, and other than variance introduced by the randomisation schedule, all animals were subject to the same protocol. The sample size was deemed sufficient to identify associations between the different treatment groups and endpoints.

**Supplemental Table 1**

Supplemental Table 1: Comparison of blood variables between groups for experiment 1. N=5-6.

| **Parameter** | Defibrillation protocol and timepoint | **N** | **Median** | **IQR** |
| --- | --- | --- | --- | --- |
| **pH** | Protocol A- Pre | 6 | 7.4 | 0.12 |
|  | Protocol B- Pre | 6 | 7.37 | 0.1 |
|  | Protocol A- 2 Hour | 6 | 7.46 | 0.03 |
|  | Protocol B- 2 Hour | 5 | 7.44 | 0.05 |
|  | Protocol A- 4 Hour | 6 | 7.49 | 0.06 |
|  | Protocol B- 4 Hour | 5 | 7.49 | 0.06 |
| **pCO_2_ (mmHg)** | Protocol A- Pre | 6 | 51.15 | 12.77 |
|  | Protocol B- Pre | 6 | 54.3 | 12.13 |
|  | Protocol A- 2 Hour | 6 | 45.8 | 2.8 |
|  | Protocol B- 2 Hour | 5 | 47.9 | 2.8 |
|  | Protocol A- 4 Hour | 6 | 47.1 | 5.15 |
|  | Protocol B- 4 Hour | 5 | 45.5 | 2.6 |
| **pO_2_ (mmHg)** | Protocol A- Pre | 6 | 238 | 89.3 |
|  | Protocol B- Pre | 6 | 245 | 19.75 |
|  | Protocol A- 2 Hour | 6 | 212.5 | 29.75 |
|  | Protocol B- 2 Hour | 5 | 221 | 29.5 |
|  | Protocol A- 4 Hour | 6 | 233 | 57.3 |
|  | Protocol B- 4 Hour | 5 | 216 | 42 |
| **BEecf (mmol/L)** | Protocol A- Pre | 6 | 5.5 | 6 |
|  | Protocol B- Pre | 6 | 5.5 | 4.75 |
|  | Protocol A- 2 Hour | 6 | 9.5 | 3.5 |
|  | Protocol B- 2 Hour | 5 | 8 | 3.5 |
|  | Protocol A- 4 Hour | 6 | 13 | 4.5 |
|  | Protocol B- 4 Hour | 5 | 11 | 3.5 |
| **HCO_3_ (mmol/L)** | Protocol A- Pre | 6 | 30.5 | 4.78 |
|  | Protocol B- Pre | 6 | 31.15 | 4.15 |
|  | Protocol A- 2 Hour | 6 | 33.25 | 2.55 |
|  | Protocol B- 2 Hour | 5 | 32.2 | 2.95 |
|  | Protocol A- 4 Hour | 6 | 36.1 | 2.73 |
|  | Protocol B- 4 Hour | 5 | 34.4 | 2.5 |
| **TCO_2_ (mmol/L)** | Protocol A- Pre | 6 | 32.5 | 5.25 |
|  | Protocol B- Pre | 6 | 33 | 4 |
|  | Protocol A- 2 Hour | 6 | 35 | 2.5 |
|  | Protocol B- 2 Hour | 5 | 34 | 3 |
|  | Protocol A- 4 Hour | 6 | 37.5 | 2.75 |
|  | Protocol B- 4 Hour | 5 | 36 | 2.5 |
| **sO_2_ (%)** | Protocol A- Pre | 6 | 100 | 0.25 |
|  | Protocol B- Pre | 6 | 100 | 0 |
|  | Protocol A- 2 Hour | 6 | 100 | 0 |
|  | Protocol B- 2 Hour | 5 | 100 | 0 |
|  | Protocol A- 4 Hour | 6 | 100 | 0 |
|  | Protocol B- 4 Hour | 5 | 100 | 0 |
| **Na (mmol/L)** | Protocol A- Pre | 6 | 138 | 4.5 |
|  | Protocol B- Pre | 6 | 137.5 | 3.25 |
|  | Protocol A- 2 Hour | 6 | 136.5 | 4.5 |
|  | Protocol B- 2 Hour | 5 | 135 | 4.5 |
|  | Protocol A- 4 Hour | 6 | 135.5 | 2.75 |
|  | Protocol B- 4 Hour | 5 | 135 | 5 |
| **K (mmol/L)** | Protocol A- Pre | 6 | 3.35 | 0.23 |
|  | Protocol B- Pre | 6 | 3.3 | 0.53 |
|  | Protocol A- 2 Hour | 6 | 4.35 | 0.7 |
|  | Protocol B- 2 Hour | 5 | 4.1 | 0.63 |
|  | Protocol A- 4 Hour | 6 | 4.35 | 0.43 |
|  | Protocol B- 4 Hour | 5 | 4.2 | 0.55 |
| **iCa (mmol/L)** | Protocol A- Pre | 6 | 1.42 | 0.1 |
|  | Protocol B- Pre | 6 | 1.48 | 0.08 |
|  | Protocol A- 2 Hour | 6 | 1.37 | 0.08 |
|  | Protocol B- 2 Hour | 5 | 1.38 | 0.11 |
|  | Protocol A- 4 Hour | 6 | 1.36 | 0.04 |
|  | Protocol B- 4 Hour | 5 | 1.35 | 0.13 |
| **Glu  (mmol/L)** | Protocol A- Pre | 6 | 39.4 | 101.5 |
|  | Protocol B- Pre | 6 | 74 | 106.1 |
|  | Protocol A- 2 Hour | 6 | 5.2 | 58.7 |
|  | Protocol B- 2 Hour | 5 | 6.2 | 63.1 |
|  | Protocol A- 4 Hour | 5 | 81 | 97.6 |
|  | Protocol B- 4 Hour | 5 | 26 | 76.9 |
| **Hct (%PCV)** | Protocol A- Pre | 6 | 19 | 5.25 |
|  | Protocol B- Pre | 6 | 21 | 4.75 |
|  | Protocol A- 2 Hour | 6 | 17.5 | 4 |
|  | Protocol B- 2 Hour | 5 | 21 | 7 |
|  | Protocol A- 4 Hour | 6 | 20 | 3.25 |
|  | Protocol B- 4 Hour | 5 | 21 | 4 |
| **Hb (via Hct) (g/dL)** | Protocol A- Pre | 6 | 6.5 | 1.78 |
|  | Protocol B- Pre | 6 | 7.15 | 1.65 |
|  | Protocol A- 2 Hour | 6 | 5.95 | 1.35 |
|  | Protocol B- 2 Hour | 5 | 7.1 | 2.35 |
|  | Protocol A- 4 Hour | 6 | 6.8 | 1.1 |
|  | Protocol B- 4 Hour | 5 | 7.1 | 1.35 |
| **WBC (×10e9/L)** | Protocol A- Pre | 6 | 15.31 | 2.49 |
|  | Protocol B- Pre | 6 | 17.4 | 2.7 |
|  | Protocol A- 2 Hour | 6 | 13.82 | 5.65 |
|  | Protocol B- 2 Hour | 5 | 15.48 | 1.43 |
|  | Protocol A- 4 Hour | 6 | 14.37 | 5.39 |
|  | Protocol B- 4 Hour | 5 | 13.75 | 2.54 |
| **Neutrophils(Segmented) (×10e9/L)** | Protocol A- Pre | 6 | 8.47 | 3.46 |
|  | Protocol B- Pre | 6 | 9.3 | 3.13 |
|  | Protocol A- 2 Hour | 6 | 6.45 | 3.9 |
|  | Protocol B- 2 Hour | 5 | 8.22 | 2.7 |
|  | Protocol A- 4 Hour | 6 | 7.9 | 4.46 |
|  | Protocol B- 4 Hour | 5 | 7.61 | 4.8 |
| **Neutrophils(segmented) (%)** | Protocol A- Pre | 6 | 57 | 15.25 |
|  | Protocol B- Pre | 6 | 55 | 11 |
|  | Protocol A- 2 Hour | 6 | 46 | 14.75 |
|  | Protocol B- 2 Hour | 5 | 58 | 15 |
|  | Protocol A- 4 Hour | 6 | 54 | 23.5 |
|  | Protocol B- 4 Hour | 5 | 50 | 27.5 |
| **Neutrophils(non-segmented) (×10e9/L)** | Protocol A- Pre | 6 | 0 | 0 |
|  | Protocol B- Pre | 6 | 0 | 0.07 |
|  | Protocol A- 2 Hour | 6 | 0 | 0 |
|  | Protocol B- 2 Hour | 5 | 0 | 0 |
|  | Protocol A- 4 Hour | 6 | 0 | 0 |
|  | Protocol B- 4 Hour | 5 | 0 | 0 |
| **Neutrophils(non-segmented) (%)** | Protocol A- Pre | 6 | 0 | 0 |
|  | Protocol B- Pre | 6 | 0 | 0.5 |
|  | Protocol A- 2 Hour | 6 | 0 | 0 |
|  | Protocol B- 2 Hour | 5 | 0 | 0 |
|  | Protocol A- 4 Hour | 6 | 0 | 0 |
|  | Protocol B- 4 Hour | 5 | 0 | 0 |
| **Lymphocytes (×10e9/L)** | Protocol A- Pre | 6 | 6.08 | 2.06 |
|  | Protocol B- Pre | 6 | 6.86 | 1.87 |
|  | Protocol A- 2 Hour | 6 | 6.79 | 2.52 |
|  | Protocol B- 2 Hour | 5 | 6.53 | 2.36 |
|  | Protocol A- 4 Hour | 6 | 5.63 | 4.05 |
|  | Protocol B- 4 Hour | 5 | 5.75 | 3.78 |
| **Lymphocytes (%)** | Protocol A- Pre | 6 | 40 | 16 |
|  | Protocol B- Pre | 6 | 41.5 | 10 |
|  | Protocol A- 2 Hour | 6 | 50.5 | 21 |
|  | Protocol B- 2 Hour | 5 | 42 | 15 |
|  | Protocol A- 4 Hour | 6 | 40 | 20 |
|  | Protocol B- 4 Hour | 5 | 49 | 26.5 |
| **Monocytes (×10e9/L)** | Protocol A- Pre | 6 | 0.15 | 0.43 |
|  | Protocol B- Pre | 6 | 0.27 | 0.41 |
|  | Protocol A- 2 Hour | 6 | 0.32 | 0.54 |
|  | Protocol B- 2 Hour | 5 | 0 | 0.08 |
|  | Protocol A- 4 Hour | 6 | 0.35 | 0.63 |
|  | Protocol B- 4 Hour | 5 | 0.14 | 0.15 |
| **Monocytes(%)** | Protocol A- Pre | 6 | 1 | 2.75 |
|  | Protocol B- Pre | 6 | 1.5 | 2.25 |
|  | Protocol A- 2 Hour | 6 | 2 | 3.25 |
|  | Protocol B- 2 Hour | 5 | 0 | 0.5 |
|  | Protocol A- 4 Hour | 6 | 2.5 | 4 |
|  | Protocol B- 4 Hour | 5 | 1 | 1 |
| **Eosinophils (×10e9/L)** | Protocol A- Pre | 6 | 0.17 | 0.17 |
|  | Protocol B- Pre | 6 | 0.16 | 0.39 |
|  | Protocol A- 2 Hour | 6 | 0 | 0.34 |
|  | Protocol B- 2 Hour | 5 | 0 | 0 |
|  | Protocol A- 4 Hour | 6 | 0.13 | 0.4 |
|  | Protocol B- 4 Hour | 5 | 0.14 | 0.21 |
| **Eosinophils(%)** | Protocol A- Pre | 6 | 1 | 1 |
|  | Protocol B- Pre | 6 | 1 | 2.25 |
|  | Protocol A- 2 Hour | 6 | 0 | 2.25 |
|  | Protocol B- 2 Hour | 5 | 0 | 0 |
|  | Protocol A- 4 Hour | 6 | 1 | 3 |
|  | Protocol B- 4 Hour | 5 | 1 | 1.5 |
| **Basophiles (×10e9/L)** | Protocol A- Pre | 6 | 0.08 | 0.22 |
|  | Protocol B- Pre | 6 | 0.09 | 0.21 |
|  | Protocol A- 2 Hour | 6 | 0 | 0.14 |
|  | Protocol B- 2 Hour | 5 | 0.14 | 0.15 |
|  | Protocol A- 4 Hour | 6 | 0 | 0.04 |
|  | Protocol B- 4 Hour | 5 | 0 | 0.14 |
| **Basophiles (%)** | Protocol A- Pre | 6 | 0.5 | 1.25 |
|  | Protocol B- Pre | 6 | 0.5 | 1.25 |
|  | Protocol A- 2 Hour | 6 | 0 | 1 |
|  | Protocol B- 2 Hour | 5 | 1 | 1 |
|  | Protocol A- 4 Hour | 6 | 0 | 0.25 |
|  | Protocol B- 4 Hour | 5 | 0 | 1 |
| **RBC (×10e12/L)** | Protocol A- Pre | 6 | 5.13 | 1.17 |
|  | Protocol B- Pre | 6 | 4.79 | 2.07 |
|  | Protocol A- 2 Hour | 6 | 4.89 | 1.31 |
|  | Protocol B- 2 Hour | 5 | 5.62 | 2.31 |
|  | Protocol A- 4 Hour | 6 | 5.57 | 1.75 |
|  | Protocol B- 4 Hour | 5 | 5.53 | 0.76 |
| **PCV (l/l)** | Protocol A- Pre | 6 | 0.26 | 0.04 |
|  | Protocol B- Pre | 6 | 0.26 | 0.04 |
|  | Protocol A- 2 Hour | 6 | 0.24 | 0.04 |
|  | Protocol B- 2 Hour | 5 | 0.28 | 0.11 |
|  | Protocol A- 4 Hour | 6 | 0.27 | 0.07 |
|  | Protocol B- 4 Hour | 5 | 0.27 | 0.03 |
| **Hb (g/dL)** | Protocol A- Pre | 6 | 8.05 | 0.95 |
|  | Protocol B- Pre | 6 | 8.3 | 0.98 |
|  | Protocol A- 2 Hour | 6 | 7.75 | 1.2 |
|  | Protocol B- 2 Hour | 5 | 8.9 | 3.35 |
|  | Protocol A- 4 Hour | 6 | 8.65 | 2 |
|  | Protocol B- 4 Hour | 5 | 8.5 | 0.85 |
| **MCV (fL)** | Protocol A- Pre | 6 | 50.55 | 7.38 |
|  | Protocol B- Pre | 6 | 50.25 | 3.3 |
|  | Protocol A- 2 Hour | 6 | 49.35 | 6.73 |
|  | Protocol B- 2 Hour | 5 | 50.2 | 4.45 |
|  | Protocol A- 4 Hour | 6 | 49.85 | 6.7 |
|  | Protocol B- 4 Hour | 5 | 49.8 | 4.4 |
| **MCHC (g/dL)** | Protocol A- Pre | 6 | 31.95 | 0.83 |
|  | Protocol B- Pre | 6 | 31.55 | 1.4 |
|  | Protocol A- 2 Hour | 6 | 32.7 | 0.73 |
|  | Protocol B- 2 Hour | 5 | 31.6 | 0.95 |
|  | Protocol A- 4 Hour | 6 | 32.25 | 0.9 |
|  | Protocol B- 4 Hour | 5 | 32.2 | 1.1 |
| **Platelets (×10e9/L)** | Protocol A- Pre | 6 | 524 | 227 |
|  | Protocol B- Pre | 6 | 596 | 175.3 |
|  | Protocol A- 2 Hour | 6 | 503.5 | 232.8 |
|  | Protocol B- 2 Hour | 5 | 452 | 213.5 |
|  | Protocol A- 4 Hour | 6 | 516.5 | 283.5 |
|  | Protocol B- 4 Hour | 5 | 593 | 156.5 |
| **RDW (%)** | Protocol A- Pre | 6 | 21.45 | 6 |
|  | Protocol B- Pre | 6 | 20.5 | 2.85 |
|  | Protocol A- 2 Hour | 6 | 19.65 | 9.5 |
|  | Protocol B- 2 Hour | 5 | 20.4 | 3.3 |
|  | Protocol A- 4 Hour | 6 | 21.6 | 5.88 |
|  | Protocol B- 4 Hour | 5 | 20.3 | 3.4 |

pCO_2_- partial pressure of carbon dioxide, pO_2_- partial pressure of oxygen, BEecf- base excess in the extracellular fluid compartment, HCO_3_- bicarbonate, TCO_2_- Total carbon dioxide, sO_2_- oxygen saturation, Na- sodium, K- potassium, iCa- ionized calcium, Glu- glucose, Hct- Hematocrit, Hb- hemoglobin, WBC- white blood cell count, RBC- red blood cell count, PCV- percentage of red blood cells in circulating blood, MCV- mean corpuscular volume, MCHC- mean corpuscular hemoglobin concentration, RDW- red blood cell distribution width. Pre- pre-intervention, 2 Hour- 2-hours post shocks, 4 Hour- 4-hours post-shocks.

**Supplemental Table 2**

Supplemental Table 2: Summary of histopathological scores for assessment of cardiac and lung tissue damage 4-hours after application of defibrillation shocks for experiment 1. There were no statistically significant differences between the groups. N=5-6.

| **Tissue sample** | **Parameter** | **Protocol** | **N** | **Median** | **IQR** | **p-value** |
| --- | --- | --- | --- | --- | --- | --- |
| **Left atria** | **Hemorrhage** | Protocol A | 6 | 0 | 0 | N/A |
|  |  | Protocol B | 5 | 0 | 0 |  |
|  | **Inflammation** | Protocol A | 6 | 0 | 0.25 | N/A |
|  |  | Protocol B | 5 | 0 | 0 |  |
|  | **Thrombosis** | Protocol A | 6 | 0 | 0 | N/A |
|  |  | Protocol B | 5 | 0 | 0 |  |
|  | **Necrosis** | Protocol A | 6 | 0.5 | 1.25 | 0.715 |
|  |  | Protocol B | 5 | 0 | 1 |  |
| **Left ventricle** | **Hemorrhage** | Protocol A | 6 | 0 | 0 | N/A |
|  |  | Protocol B | 5 | 0 | 0 |  |
|  | **Inflammation** | Protocol A | 6 | 0 | 0 | N/A |
|  |  | Protocol B | 5 | 0 | 0 |  |
|  | **Thrombosis** | Protocol A | 6 | 0 | 0 | N/A |
|  |  | Protocol B | 5 | 0 | 0 |  |
|  | **Necrosis** | Protocol A | 6 | 1.5 | 2.25 | 0.523 |
|  |  | Protocol B | 5 | 1 | 1.5 |  |
| **Right atria** | **Hemorrhage** | Protocol A | 6 | 0 | 0 | N/A |
|  |  | Protocol B | 5 | 0 | 0 |  |
|  | **Inflammation** | Protocol A | 6 | 0 | 0 | N/A |
|  |  | Protocol B | 5 | 0 | 0 |  |
|  | **Thrombosis** | Protocol A | 6 | 0 | 0 | N/A |
|  |  | Protocol B | 5 | 0 | 0 |  |
|  | **Necrosis** | Protocol A | 6 | 0 | 1 | 0.927 |
|  |  | Protocol B | 5 | 0 | 1 |  |
| **Right ventricle** | **Hemorrhage** | Protocol A | 6 | 0 | 0 | N/A |
|  |  | Protocol B | 5 | 0 | 0 |  |
|  | **Inflammation** | Protocol A | 6 | 0 | 0 | N/A |
|  |  | Protocol B | 5 | 0 | 0 |  |
|  | **Thrombosis** | Protocol A | 6 | 0 | 0 | N/A |
|  |  | Protocol B | 5 | 0 | 0 |  |
|  | **Necrosis** | Protocol A | 6 | 0 | 1.25 | 0.855 |
|  |  | Protocol B | 5 | 0 | 1 |  |
| **Lung** | **Hemorrhage** | Protocol A | 6 | 0 | 0 | N/A |
|  |  | Protocol B | 5 | 0 | 0.5 |  |
|  | **Inflammation** | Protocol A | 6 | 0 | 0 | N/A |
|  |  | Protocol B | 5 | 0 | 0.5 |  |
|  | **Thrombosis** | Protocol A | 6 | 0 | 0 | N/A |
|  |  | Protocol B | 5 | 0 | 0 |  |
|  | **Necrosis** | Protocol A | 6 | 0 | 0.00 | N/A |
|  |  | Protocol B | 5 | 0 | 0 |  |
